# Development of an Artificial Neural Network as a Tool for Predicting the Targeted Phenolic Profile of Grapevine (*Vitis vinifera*) Foliar Wastes

**DOI:** 10.3389/fpls.2018.00837

**Published:** 2018-06-19

**Authors:** Maliheh Eftekhari, Abbas Yadollahi, Hamed Ahmadi, Abdolali Shojaeiyan, Mahdi Ayyari

**Affiliations:** ^1^Department of Horticultural Sciences, Faculty of Agriculture, Tarbiat Modares University, Tehran, Iran; ^2^Bioscience and Agriculture Modeling Research Unit, College of Agriculture, Tarbiat Modares University, Tehran, Iran

**Keywords:** bioactive compounds, grapevine waste, neural network, prediction, regression

## Abstract

High performance liquid chromatography data related to the concentrations of 12 phenolic compounds in vegetative parts, measured at four sampling times were processed for developing prediction models, based on the cultivar, grapevine organ, growth stage, total flavonoid content (TFC), total reducing capacity (TRC), and total antioxidant activity (TAA). 12 Artificial neural network (ANN) models were developed with 79 input variables and different number of neurons in the hidden layer, for the prediction of 12 phenolics. The results confirmed that the developed ANN-models (*R*^2^ = 0.90 – 0.97) outperform the stepwise regression models (*R*^2^ = 0.05 – 0.78). Moreover, the sensitivity of the model outputs against each input variable was computed by using ANN and it was revealed that the key determinant of phenolic concentration was the source organ of the grapevine. The ANN prediction technique represents a promising approach to predict targeted phenolic levels in vegetative parts of the grapevine.

## Introduction

Grapevine (*Vitis vinifera*) as one of the most economically important fruit crops worldwide, has been extensively cultivated all over the world for fresh consumption as well as industrial processing. Both cultivation and industrial processing of grapes lead to byproducts, such as leaves and stems which have been found as the enriched resources of bioactive polyphenolic compounds (Eftekhari et al., 2017). The phenolics worth is due to their important roles in protection against degenerative diseases such as cancer and atherosclerosis, in addition to antimicrobial, anti-inflammatory, antidiabetic and skin protection, hepatoprotective, and neuroprotective activities (Nassiri-Asl and Hosseinzadeh, 2016). Extracts prepared from grapevine leaves and stems are known to be promising sources of grape polyphenols including flavonoids, phenolic acids, stilbenes and COU. These compounds have recently received considerable attention, and because of their pharmacological impacts and antioxidant activity (Xia et al., 2010) can be used as natural antioxidants in the pharmaceutical, food and cosmetic industries (Houillé et al., 2015; Torres et al., 2015).

In the standard analytical approach, the identification and quantification of phenolic compounds requires expensive and complex equipment including high performance liquid chromatography (HPLC) and expensive pure standards. An alternative approach, using simple measurements to predict the phenolic profile in each part of every grapevine cultivar at defined developmental stages offers a substitute for the use of foliar waste materials. This prediction may prevent the loss of high enriched valuable sources of phenolics. Finding the association between easier and lower cost measuring indexes such as TRC, TFC, and TAA with individual phenolics content can be a supplementary source to predict the level of important phenolics in above mentioned wastes. ANN modeling provides a way of analyzing uneven and multi-dimensional datasets arising from such data collection activities. Datasets are used by software models to construct networks.

The data set is divided into training and test data. With the large number of connections, ANN can find the key non-linear relationships between the determined input and corresponding output variables in the training dataset used to develop the model, and then apply that knowledge to new inputs from previously untested samples (Hashimoto, 1997; Guoqiang et al., 1998). Accordingly, ANN look for a mathematical formulation in the training dataset used for model development to achieve the closest result to the expected value. The ANN technique is especially helpful for complicated problems involving numerous variables with restricted knowledge of the interactions between variables and their variation (Suárez et al., 2015). Hence, considering the importance of predicting phenolic contents in agricultural wastes without expensive analyses, this study aims to evaluate and validate accuracy of the ANN technique to predict phenolics levels and composition in grapevine vegetative parts as waste material of pruning and other viticultural practices due to its capability of learning complex, non-linear relationships between the input and output.

Based on ours and other published works (Houillé et al., 2015; Torres et al., 2015; Eftekhari et al., 2017), it has been shown that the vegetative parts of grapevines, which are often waste-products in grape cultivation and production, are rich in phenolics. Their phenolic composition is determined primarily by genetic factors and the identity of the organ (leaf or stem) (Eftekhari et al., 2017), and it may change during the vine development (Teixeira et al., 2013; Eftekhari et al., 2017). Meanwhile, the influence of additional factors remains unknown, as does prediction of the phenolics profile in different organs in various time points through the vine annual growth cycle.

This study first presents and compares the stepwise regression analyses for 12 important phenolic compounds to determine the importance of each factor in determining the level of the phenolics and find the association between them. A back-propagation ANN model, which consists of cultivar, organ, sampling time, TRC, TFC and TAA was established for predicting each phenolic compound using the Matlab software. The data were obtained from HPLC for individual phenolics and spectrophotometry for TRC, TFC, and TAA. The total measurements reflected the phenolic properties of the sample; but if they have a direct relationship with individual phenolics is still a question which have been analyzed only with correlation analysis in the literature (Karacabey et al., 2012; Eftekhari et al., 2017). By training the network with specified inputs and outputs, we predict the targeted phenolics content of grape cultivars foliar parts. And then the trained network can make predictions for *V. vinifera* cultivars considering the same factors. Cultivar, organ, time, as well as TFC, TRC and TAA were set as input variables, while each phenolic content was set as output to separate ANN networks. Then, the best network to predict output was selected based upon an optimization procedure using a GA. Finally, for the purpose of performance comparison, the predicted results of ANN models related to 12 phenolic compounds were compared to the respective stepwise regression models. The goodness of fit of the ANN and regression models were assessed using statistical analysis.

To our knowledge, the research described here represents for the first time, the use of artificial neural network prediction technique for predicting phenolic contents in plant material. Our results provide an important contribution to this research area and industrial field. To promote the commercial consumption of these bioactive plant materials, it is important to predict the phenolic potential of these grapevine leftovers in a special cultivar and organ in a particular time.

## Materials and Methods

### Samples and Datasets

In a previous study (Eftekhari et al., 2017), we collected leaves and stems from 5-year-old vines of 70 Iranian native grape cultivars [Supplementary Table S1 in supplementary material of Eftekhari et al. (2017)] growing in the Research Farm of Faculty of Agriculture, University of Tehran, Karaj, Alborz, Iran (latitude 35° 50′ N, longitude 50° 58′ E and altitude 132 m) at the middle of 4 months of July to October 2015. Collected samples were extracted after oven-drying at 40°C for 72 h, using the method described by Eftekhari et al. (2012).

Extracts were analyzed for their phenolic composition: flavonoids CAT, KAE, QUE, RUT, NAR, and IQ, phenolic acids GAL, PC, OC, and MC, stilbene RES and COU.

Beginning with the measured analytical data of HPLC we used a data matrix containing the results of the samples coming from different cultivars and organs (leaves or stems) at different times (July–October), to test the capability of ANN method in modeling the targeted phenolic profiling of *V. vinifera* foliage in relation to cultivar, organ and harvest time. A factorial design arrangement resulted in the total 1890 data (**Table 1**).

**Table 1:** Levels of variables according to the factorial arrangement of 1890 data related to phenolic compounds concentrations measured by HPLC.

Sample	Cultivar^a^	Organ^b^	Time^C^	TRC^d^	TFC^e^	TAA^f^	GAL^g^	CAT	PC	RUT	IQ	MC	OC	COM	RES	QUE	NAR	KAE
**Training data**																		
**1**	1	1	1	3340.9	10020.5	10573.9	58.4	81.7	37.7	599.1	209.2	42.7	137.7	1141.1	142.4	74.5	0.0	0.0
**2**	1	1	1	2338.3	10031.3	10332.5	61.4	88.3	38.1	590.5	214.0	42.6	142.7	1148.5	142.0	72.5	0.0	0.0
**3**	1	1	2	6548.8	8550.8	9420.6	58.0	2969.0	94.2	560.6	274.5	48.9	413.3	1156.9	142.4	315.7	0.0	33.5
**4**	1	1	2	6521.3	8548.4	9392.1	59.0	3282.9	72.1	604.2	296.7	51.4	291.4	1252.8	152.7	349.5	0.0	33.3
**5**	1	2	2	33263.6	1101.8	102571.5	0.0	0.0	0.0	0.0	0.0	0.0	0.0	238.7	0.0	516.1	0.0	0.0
**6**	1	2	2	32379.0	1046.9	103011.3	0.0	0.0	0.0	0.0	0.0	0.0	0.0	246.7	0.0	527.1	0.0	0.0
**7**	1	2	3	2331.3	309.5	8491.6	0.0	48.2	0.0	0.0	22.3	24.2	0.0	0.0	34.6	73.1	0.0	0.0
**8**	1	2	3	2408.6	303.5	8361.2	0.0	48.0	0.0	0.0	22.9	24.8	0.0	0.0	29.5	75.6	0.0	0.0
**9**	1	2	3	2369.9	306.5	8426.4	0.0	48.1	0.0	0.0	22.6	24.5	0.0	0.0	32.0	74.4	0.0	0.0
**10**	1	1	4	10092.7	10377.4	11029.5	93.3	1022.5	104.8	793.8	1004.3	71.2	9604.4	80.5	67.2	357.0	37.2	158.7
**11**	1	1	4	6458.8	10752.3	10833.8	89.9	1007.7	105.4	811.6	1024.1	71.8	9778.3	82.7	68.7	372.6	38.9	109.0
**12**	1	1	4	8275.8	10564.9	10931.6	91.6	1015.1	105.1	802.7	1014.2	71.5	9691.3	81.6	68.0	364.8	38.1	133.9
**13**	1	2	4	976.3	1329.4	9163.6	0.0	38.5	0.0	0.0	0.0	0.0	0.0	0.0	0.0	60.6	0.0	0.0
**14**	1	2	4	2487.0	1419.0	9076.3	0.0	38.5	0.0	0.0	0.0	0.0	0.0	0.0	0.0	60.6	0.0	0.0
**15**	2	1	1	4347.2	10412.6	10498.6	72.2	130.9	50.8	956.4	220.3	36.1	126.9	1384.7	113.7	95.8	0.0	0.0
**16**	2	1	1	5571.5	10326.2	11585.8	72.9	134.1	50.6	964.8	229.7	38.7	394.7	1440.7	119.6	99.8	0.0	0.0
**17**	2	2	2	24965.2	1406.6	104444.9	0.0	0.0	0.0	0.0	203.6	0.0	0.0	230.3	0.0	503.5	0.0	0.0
**18**	2	1	3	1260.1	13656.5	12302.3	60.2	1852.4	42.4	119.4	45.0	29.2	6336.2	80.1	47.4	95.2	0.0	0.0
**19**	2	1	3	1111.3	13486.3	12582.2	60.5	2058.3	45.0	125.8	22.6	29.7	6908.3	86.5	49.4	99.2	0.0	32.3
**20**	2	2	3	2659.8	39.1	8291.4	0.0	60.9	0.0	0.0	24.0	0.0	0.0	0.0	47.9	72.0	0.0	0.0
**21**	2	2	3	2626.3	48.8	8196.3	0.0	54.4	0.0	0.0	22.8	0.0	0.0	0.0	43.8	68.3	0.0	0.0
**22**	2	1	4	14007.8	10370.0	11521.0	123.1	3607.1	135.8	1429.9	1102.5	77.0	2551.4	93.3	96.4	830.3	47.0	370.0
**23**	2	1	4	15739.3	10501.8	11420.1	101.8	2784.1	114.7	1180.9	932.3	68.0	1434.1	81.8	116.3	717.8	45.8	327.0
**…**	…	…	…	…	…	…	…	…	…	…	…	…	…	…	…	…	…	…
**Testing data**																		
**1229**	59	1	2	1795.4	10518.1	10351.8	101.9	231.1	54.1	831.1	170.3	348.5	617.8	80.0	68.0	11337.0	100.8	366.8
**1230**	39	1	4	5393.4	10584.1	10966.1	90.1	438.0	126.9	372.1	47.1	64.6	3711.7	37.5	2168.6	236.2	52.8	203.8
**1231**	67	1	4	7185.9	10557.0	10146.9	102.0	1762.5	98.3	904.3	219.8	261.5	253.1	0.0	207.5	397.4	48.9	189.6
**1232**	70	1	4	12013.9	14094.8	14572.4	149.7	6225.3	690.6	1477.2	485.2	243.0	6325.8	0.0	291.3	533.0	80.2	174.2
**1233**	21	1	3	4991.5	10275.7	10304.2	82.8	193.3	1192.3	1190.2	48.5	60.1	700.6	259.5	125.9	13123.2	12.8	156.6
**1234**	22	1	3	1192.2	10510.0	9755.9	88.6	112.4	879.9	1071.7	40.0	43.4	1709.6	164.8	45.5	18220.8	0.0	139.9
**1235**	59	1	4	6001.2	10592.6	9477.2	96.3	698.8	188.6	872.9	282.8	268.7	1477.5	1110.7	266.8	661.8	49.7	118.7
**…**	…	…	…	…	…	…	…	…	…	…	…	…	…	…	…	…	…	…


*^*a*^Cultivars codes according to the Supplementary Table S1 in supplementary material of*Eftekhari et al. (2017). *^*b*^Organ 1 = leaf, 2 = stem. ^*C*^Time of harvest: 1 = July, 2 = Agust, 3 = September, 4 = October. ^*d*^Total flavonoid content (mg QE/kg dw). ^*e*^Total reducing capacity (mg GAE/kg dw). ^*f*^Total antioxidant activity (mg AAE/kg dw). ^*g*^mg/kg dw of GAL, Gallic acid; CAT, catechin; PC, p-coumaric acid; RUT, rutin; IQ, isoquercitrin; MC, m-coumaric acid; OC, o-Coumaric acid; COU, coumarin; RES, resveratrol; QUE, quercetin; NAR, naringenin; KAE, kaempferol.*

### Statistical Analysis and Model Development

#### Stepwise Regression Modeling

The popularity of regression models lie in their ease of use and interpretation. A multiple regression model with more than one explanatory variable may be written as *y* = *b*_0_ + *b*_i_*X*_i_ + … + *b*_p_*X*_p_, where y is the output variable, b the regression coefficient (*i* = 0, 1, 2,…, p) with *b*_0_ as the intercept, and *X* the input variable (*i* = 1, 2,…, p). When regression coefficients are attained, an equation of prediction can then be applied to forecast the continuous outputs as linear functions of independent inputs. The regression models popularity may be due to the interpretability of model parameters coefficients and simplicity of use. Here, for the prediction of phenolic composition, the forward stepwise regression models are used and the entry and stay levels of the *p*-values were set at 0.05 for the models. Independent variables were selected according to the maximum *F*-value if the associated partial correlation coefficient is zero:

$$F=\frac{\left(n-q-2\right)r_{yx(q+1)\cdot x\left(q\right)}^{2}}{1-r_{yx(q+1)\cdot x\left(q\right)}^{2}}$$

where r_yx(*q*+1).*x*(*q*)_ is the sample partial correlation coefficient between *y* and the selected (*q*+1) independent variable with *q* as the selected independ variable, the related *P*-value is:

$$P=\mathrm{Pr}\left[F>F_{\max}\right]$$

Whenever *P* exceeds the defined α level, stopping occurs.

The stepwise regression analyses were performed on the data to test significance of the independent variables cultivar, organ, harvest time, TRC, TAA, and TFC affecting level of phenolics GAL, CAT, PC, RUT, IQ, MC, OC, COU, RES, QUE, NAR, and KAE in grapevine foliage as dependent variables. The regression analyses were performed using SAS 9.1 (SAS Institute, Cary, NC, United States).

#### Hybrid ANN-GA Modeling Procedure

ANN-GA procedures are adaptive having the parallel information-processing structures, which are able to make functional associations between data and to provide predominant tools for non-linear, multidimensional incorporations. Back-propagation is an optimization algorithm applied on ANNs to minimize the training error function by iteratively adjusting the weights and biases of the ANN which comprises three adjacent layers called the input, hidden and output layers which may have a number of sub-layers. Each layer consists of a certain number of neurons that needs to be optimized. ANNs are prevailing tools for estimation of unknown non-linear functions and have extensive applications in various fields (Rumelhart and McClelland, 1986; Lawrence, 1994).

**FIGURE 1:**
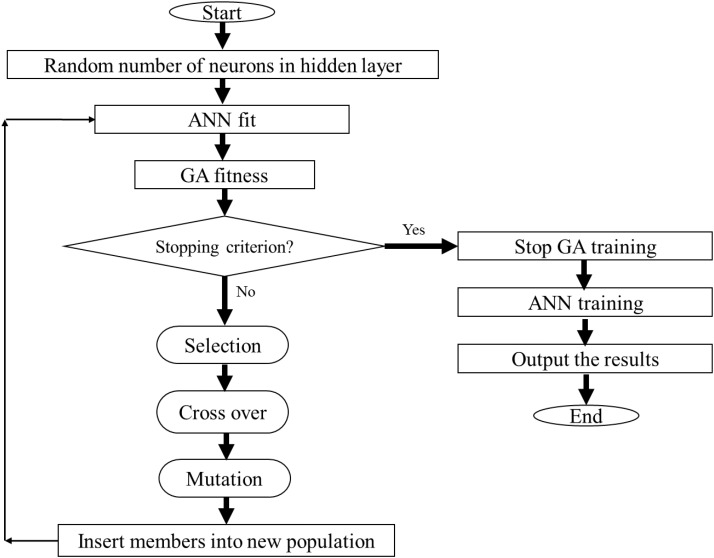
Flow chart of integrating ANN with GA for optimization of inputs combination to achieve the highest amount of each metabolite.

In this study, the hybrid ANN-GA strategy (Mirarab et al., 2014) was used to efficiently optimize the neuron number in the hidden layer (Wang, 2005). The structure of the ANN-GA used in this paper is shown in **Figure 1**. We built a three-layer feed-forward ANN and applied the backpropagation algorithm to obtain the best fit to the training data because of its capacity of representing non-linear functional relationships between inputs and targets. As activation functions, we used the hyperbolic tangent sigmoid (tansig) for the hidden layer and linear (purelin) for the output layer. The Levenberg-Marquardt back-propagation training algorithm was used for minimizing the error function of the ANN (Beale et al., 2010). The fitness performance function of the GA was used to determine the optimum structure of the ANN (**Figure 1**). The mean square error (MSE) was used as performance function and learning was completed after 800 epochs. Three replicates of each treatment resulted in a dataset with total of 1890 observations. These data were randomly divided into two distinct groups: 65% of data lines (1228 data) for training set and, 35% (662 data) for testing set. The same set of data were used for developing 12 neural network models related to 12 phenolic compounds. Using replicates instead of mean values in ANN modeling helps to assess not only the mean, but also the range of their deviation in the model (Silva et al., 2015). The training set was used to compute all the parameters of the ANN and the testing set was used to evaluate the precision of the neural network prediction.

Furthermore, the GA was used in order to determine the optimal neuron numbers in the hidden layer (**Figure 1**). So, elite populations were selected for crossover using a roulette wheel selection method. An initial population of 50, generation number of 500, crossover rate of 0.85, and mutation rate of 0.01 were used to obtain the best ANN structure (Haupt and Haupt, 2004; Abramson, 2007). The stop criterion was on the basis of the MSE with the lowest level as the network performance function for the training dataset (**Figure 1**). To select the best ANN structures with optimum neuron number in the hidden layer, the total number of 3 to 10 neurons were used based on GA. High number of hidden neurons may cause overlearning of the neural network. On the other hand, too few hidden neurons will lead to under fitting. The generational action was frequently run to attain the number of generations.

The sensitivity analysis was performed on the developed ANN-models to find the importance of the input variable (cultivar, organ, time, TRC, TFC, and TAA) in the model for determining the content of phenolics CAT, KAE, QUE, RUT, NAR, IQ, GAL, PC, OC, MC, RES, and COU in grapevine foliar parts. The sensitivity is determined as the ratio between the error of the eliminated variable and the baseline error. It ranks the variables according to their importance and determines which variables that can be omitted and which variables that are important to keep in further analyses. The sensitivity of phenolics content against cultivar, organ, time, TRC, TFC, and TAA was determined using the criteria (Lou and Nakai, 2001; Ahmadi and Golian, 2010a,b) as follows: the variable sensitivity error (VSE) value is computed as the performance of the ANN-model where that variable is omitted, and the estimate of the variable sensitivity ratio (VSR) is then calculated as the relative ratio between the VSE and the error of an ANN-model where all variables are included. Higher VSR value shows more important variable. So, the input variables may be ranked in the order of importance on the basis of the obtained VSR value.

Matlab R2010a (Matlab R2010a, 2010) software was applied for writing mathematical code to build and evaluate the ANN-models. In fact, the developed program is a modified source code of an ANN algorithm which was previously used by (Ahmadi and Golian, 2011; Arab et al., 2016; Jamshidi et al., 2016).

Twelve models were developed separately for predicting the concentration of CAT, KAE, QUE, RUT, NAR, IQ, GAL, PC, OC, MC, RES, and COU. In order to make the model computationally more tractable, both the input and output data were normalized to the range of -1 to 1 (Beale et al., 2010; Gulati et al., 2010; Ahmadi and Golian, 2011).

For determining the accuracy of the developed ANN and regression models, the statistical parameters root mean square error (RMSE) and mean bias error (MBE) were applied in addition to *R*^2^, using the following formulas (Ahmadi et al., 2007):

R2=1−Σi=1n(y^−y)2Σi=1n(y−y¯)2

RMSE=(Σi=1n(y^−y)2)/n

MBE=1/n⁢ Σi=1n|y^−y|

where *n* is the number of observations in the test data, *y* are the values of the output in the test data, and y are values of the predicted outputs.

## Results

### Stepwise Regression Modeling

According to the results of the stepwise regression models (**Table 2**) (number of observations = 1890), organ, time and cultivar were found to differently affect the phenolics content. So that RUT and NAR concentrations are under the effect of all three factors while for GAL, *m*- and OCs and COU levels, cultivar is not an important factor as well as time which is not critical in determining IQ, RES and PC content. Cultivar is the only factor among three above mentioned factors which affects QUE level whereas time is important for CAT and KAE content.

**Table 2:** Stepwise regression model of cultivar, organ (leaf or stem), month (July, August, September, and October), TRC, TFC, and TAA for different measured phenolics content of *V. vinifera* foliar^a^.

Measured factor	Variable^a^	Parameter estimation	Standard error
Gallic acid	**Intercept**	111.45986	6.17210
	**Organ**	-67.80296	3.03427
	**Month**	6.33617	0.60438
	**TRC**	0.00043	0.00006
	**TFC**	0.00079	0.00032
*R*^2^		0.78380	
RMSE^b^		16.87642	
MBE^C^		8.90901	
Catechin			
	**Intercept**	690.33232	138.70621
	**Month**	-215.99405	34.93546
	**TAA**	-0.01073	0.00180
	**TRC**	0.02550	0.00589
	**TFC**	0.08383	0.00589
*R^2^*		0.20350	
RMSE		888.37214	
MBE		546.28009	
*p*-coumaric acid			
	**Intercept**	269.28027	15.44732
	**Organ**	-115.13223	9.21706
	**Cultivar**	-1.07779	0.20843
	**TRC**	0.00163	0.00037
*R*^2^		0.19460	
RMSE		101.06410	
MBE		61.11106	
Rutin			
	**Intercept**	776.67442	35.50774
	**Organ**	-407.04701	21.12686
	**TRC**	0.00998	0.00148
	**Cultivar**	-2.21759	0.41505
	**Month**	31.43606	9.05637
	**TAA**	-0.00105	0.00048
*R*^2^		0.33550	
RMSE		223.31979	
MBE		156.03616	
Isoquercitrin			
	**Intercept**	447.57991	20.82450
	**Organ**	-200.06385	12.73737
	**TRC**	0.00743	0.00096
	**Cultivar**	-1.36077	0.26954
	**TAA**	-0.00130	0.00029
*R*^2^		0.2796	
RMSE		124.35464	
MBE		94.42968	
*m*-coumaric acid			
	**Intercept**	104.20099	7.86134
	**Organ**	-63.64240	4.62065
	**TRC**	0.00163	0.00020
	**Month**	9.08716	2.04670
*R*^2^		0.11200	
RMSE		64.32313	
MBE		39.04541	
*o*-coumaric acid			
	**TFC**	0.04527	0.01659
	**Month**	390.38893	31.47157
	**TRC**	0.02274	0.00313
	**Organ**	-739.79659	158.00088
*R*^2^		0.17610	
RMSE		820.62908	
MBE		399.13679	
Coumarin			
	**Intercept**	1526.68644	59.78865
	**Organ**	-462.00556	39.17702
	**Month**	-170.17934	16.81054
	**TAA**	-0.00382	0.00088
	**TRC**	0.00547	0.00275
*R*^2^		0.47530	
RMSE		382.57347	
MBE		246.86150	
Resveratrol			
	**Intercept**	436.31941	50.74028
	**Organ**	-235.66322	27.79515
	**Cultivar**	1.47387	0.69026
*R*^2^		0.05860	
RMSE		307.85297	
MBE		110.67512	
Quercetin			
	**TFC**	0.10599	0.01387
	**Cultivar**	-7.02131	3.12538
*R*^2^		0.04740	
RMSE		1322.70590	
MBE		535.04881	
Naringenin			
	**Intercept**	15.29345	2.26374
	**Organ**	-19.70467	1.20828
	**Month**	6.36006	0.53486
	**TRC**	0.00038	0.00005
	**Cultivar**	0.07100	0.02650
*R*^2^		0.21260	
RMSE		11.72686	
MBE		7.69754	
Kaempferol			
	**Intercept**	-23.91979	5.30187
	**Month**	9.96098	1.70531
	**TFC**	0.00412	0.00042
*R*^2^		0.12590	
RMSE		32.28414	
MBE		22.06913	


*^*a*^Significant (p < 0.05) variables left in the model. ^*b*^RMSE, root mean square error, ^*c*^MBE, mean bias error*.

The amounts of TRC, TFC, and TAA showed various relationships with the content of phenolics, as well. As CAT is associated with all three measured factors but TAA is excluded about GAL and OC as well as exclusion of TFC for RUT, IQ, and COU. NAR, *p*- and MCs are just related to TRC while QUE and KAE are associated to TFC. RES content did not show any relationship with the last three measured factors.

### ANN-GA Modeling and Sensitivity Analysis

To verify the capability of the ANN in phenolic profiling prediction, we used empirical data of our previous HPLC analyses (Eftekhari et al., 2017). Initially, we used cultivar, organ, sampling time, TRC, TFC, and TAA as input variables and each phenolic concentration as the output variable of the network, and the concentration of every phenolic compound could be predicted according to the trained network in separate models.

In order to test the performance of the developed ANN-models, the predicted and experimental datasets of training samples were compared and the results presented in **Table 3** show the high ability of the ANN to produce outputs close to the experimental data. The average accuracy (*R*^2^ = 0.95) of training data is indicating that the developed network could be used for testing data in the subsequent analysis.

**Table 3:** Statistics and information on artificial neural network models for measured phenolics in leaf and stem extracts of *V. vinifera* during 4 months (training vs. testing values).

Compound		*R*^2^	RMSE^a^	MBE^b^
Gallic acid	Training	0.9937	3.17	1.27
	Testing	0.9723	6.02	1.97
Catechin	Training	0.9615	228.41	93.25
	Testing	0.9176	283.87	118.06
*p*-coumaric acid	Training	0.9674	25.53	9.58
	Testing	0.9257	30.76	14.44
Rutin	Training	0.9695	56.42	33.19
	Testing	0.9228	75.21	42.16
Isoquercitrin	Training	0.9684	35.30	20.08
	Testing	0.9179	41.07	25.41
*m*-coumaric acid	Training	0.9361	17.51	9.04
	Testing	0.9196	19.38	11.39
*o*-coumaric acid	Training	0.9801	159.72	53.93
	Testing	0.9247	251.31	84.27
Coumarin	Training	0.9405	142.92	49.21
	Testing	0.9129	156.44	62.26
Resveratrol	Training	0.9114	133.30	37.72
	Testing	0.9179	95.88	47.30
Quercetin	Training	0.9315	526.26	168.05
	Testing	0.9606	275.36	146.74
Naringenin	Training	0.9495	4.35	1.79
	Testing	0.9027	3.92	1.96
Kaempferol	Training	0.90	20.48	8.65
	Testing	0.903	10.37	6.57


*^*a*^RMSE, root mean square error; ^*b*^MBE, mean bias error.*

In order to evaluate the generalization capability of the model, we examined the response ability of established models to respond to the testing dataset not involved in the training process. The prediction results of the testing dataset are listed in **Table 3**. Clearly, a high correlation between the predicted results and targets is noticeable. The average testing accuracy (*R*^2^ = 0.92) is indicating that the developed network is efficient and feasible.

The error statistics evaluated on our developed ANN-models are highly constant for both training and test data prediction of each output (**Table 3**) suggesting lack of over fitting throughout the training process (Ahmadi and Golian, 2010a,b).

In order to determine the relative importance of input variables, the entire dataset was used to estimate the overall VSR for each phenolic concentration. The obtained VSR for each output variable of the models concerning 79 input variables are shown in **Table 4**.

**Table 4:** Importance of evaluated factors on phenolics content of grapevine vegetative parts according to the sensitivity analysis on the developed neural network models.

Element	VSR^a^					
	
	Organ	Month	Cultivar	TRC	TFC	TAA
Gallic acid	57.00	22.95	21.54	15.55	13.04	2.37
Rank	1	2	3	4	5	6
Catechin	77.62	23.30	19.84	5.68	1.88	1.69
Rank	1	2	3	4	5	6
*p*-coumaric acid	23.34	17.98	12.94	2.19	2.17	2.02
Rank	1	2	3	4	5	6
Rutin	88.00	36.63	35.95	9.36	5.67	1.88
Rank	1	2	3	4	5	6
Isoquercitrin	34.04	17.36	14.92	2.31	1.10	1.33
Rank	1	2	3	4	6	5
*m*-coumaric acid	15.62	9.83	7.71	1.42	1.32	1.28
Rank	1	2	3	4	5	6
*o*-coumaric acid	27.64	14.70	10.99	1.73	1.45	3.35
Rank	1	2	3	5	6	4
Coumarin	19.77	11.66	7.46	1.60	1.37	1.26
Rank	1	2	3	4	5	6
Resveratrol	41.27	15.17	10.65	3.27	1.28	1.21
Rank	1	2	3	4	5	6
Quercetin	39.54	16.33	11.76	1.08	4.70	1.16
Rank	1	2	3	6	4	5
Naringenin	21.37	8.46	6.91	1.20	2.33	1.03
Rank	1	2	3	5	4	6
Kaempferol	68.86	10.78	7.03	1.43	3.48	1.20
Rank	1	2	3	5	4	6


*^*a*^Relative indication of the ratio between the variable sensitivity error and the error of the model when all variables are available.*

The higher the VSR value, the more important is the input variable. Thus, the inputs can be ranked according to their importance in determining the outputs using VSR values (**Table 4**).

Among the input variables, organ had the highest values of VSR in datasets for all phenolics. According to the obtained VSR values, the order of the most important phenolics in grapevine were organ, time, cultivar, TRC, TFC, and TAA, respectively. But about the last three mentioned factors, OC was more sensitive to TAA (3.35) followed by TRC (1.73) and TFC (1.45), while QUE was more sensitive to TFC (4.70) followed by TAA (1.16) and TRC (1.08) (**Table 4**). The order of input sensitivity for NAR and KAE was as TFC (2.33 and 3.48), TRC (1.20 and 1.43) and TAA (1.03 and 1.20).

### Comparison of ANN-GA and Stepwise Regression Models

The estimated statistical values related to the ANN-models revealed a substantially higher accuracy of prediction than for regression models, so as calculated *R*^2^ for ANN vs. regression models were: GAL = 0.97 vs. 0.78, CAT = 0.92 vs. 0.19, PC = 0.93 vs. 0.18, RUT = 0.92 vs. 0.34, IQ = 0.92 vs. 0.27, MC = 0.92 vs. 0.11, OC = 0.92 vs. 0.18, COU = 0.91 vs. 0.48, RES = 0.92 vs. 0.06, QUE = 0.96 vs. 0.05, NAR = 0.90 vs. 0.21 and KAE = 0.90 vs. 0.13 (**Tables 2**, **3**). In order to develop an accurate prediction model, it is important to use a reliable modeling system to predict subjects.

### Accuracy of ANN Prediction

Based on the train and test accuracies (**Table 3**), we can conclude that the use of the tansig activation function provides a rational choice for modeling non-linearities over all experiments. The number of neurons in hidden layer (**Table 5**) as well as close errors of training and testing subsets ensure that over-learning has not happened (Matlab R2010a, 2010). The strength of our work is that we used the same datasets (training and testing) for developing different ANN-models which confirms that the developed models are quite reliable and valid.

**Table 5:** Structure of artificial neural networks used to build models for prediction of phenolics concentrations.

Phenolic compound	Number of input layer(s)	Number of hidden layer neurons	Number of output layer(s)
Gallic acid	79	7	1
Catechin	79	8	1
*p*-coumaric acid	79	8	1
Rutin	79	6	1
Isoquercitrin	79	9	1
*m*-coumaric acid	79	8	1
*o*-coumaric acid	79	8	1
Coumarin	79	7	1
Resveratrol	79	6	1
Quercetin	79	7	1
Naringenin	79	10	1
Kaempferol	79	7	1


As it was pointed out before, plant organ is of critical importance since phenolics accumulate in different organs according to the plant growth stage (Eftekhari et al., 2017) and the content of phenolic compounds is also cultivar dependent (Eftekhari et al., 2017). Predicting the amount of phenolics in different parts of the grapevine as enriched raw material for extraction and industrial applications is highly helpful and much required as knowledge of the phenolic profiles and features of the samples will assist to make a decision in the collection of the most appropriate sample for industrial scale extraction increasing the value of a potential commercial product.

## Discussion

To the best of our knowledge, this study is the first to give an idea about the prediction of phenolic composition in grapevine foliage. Using the combination of ANN and GA is recommended as a promising prediction method to evaluate phenolics in different grape cultivars, organs and developmental stages. And this technique cannot only be useful for making predictions for high value bioactive compounds, but also provides new potential approaches for bio-compounds research into other plants and other environmental conditions.

The present study was conducted to predict the phenolic profiles of grapevine (*V. vinifera*) leaves and stems, as residues of the viticulture or winery industries, addressed to food, pharma or cosmetic industries. Previous results (Eftekhari et al., 2017) on the *V. vinifera* cultivars foliar parts revealed that they contain high levels of valuable phenolic compounds comparable or more than the reported levels in different parts of the fruit and winery by-products (Di Lecce et al., 2014; Ky et al., 2014). In the same study, grape cultivars were discriminated according to the phenolic compounds composition in their foliar parts during grapevine development confirming the significant impact of vine growth stage in addition to cultivar and organ on phenolics accumulation. Recent valorization studies on grape and wine industry wastes open paths to the production of bioactive compounds. Various analytical spectrophotometric techniques have been established based on different principles for the total determination of different structural groups existing in the phenolic compounds such as TFC. These determinations accompanied by the antioxidant activity are preliminary evaluation of polyphenol content due to providing valuable information about the comparative content and potential bioactivity of the sample (Cámara et al., 2010; Fontana et al., 2017).

The diverse phytochemical contents of leaves and stems (Eftekhari et al., 2017) together with the drive to reduce environmental effects on wastes has led to viticulture waste valorization initiatives including using those wastes as a source for the production of high-value bioactive chemicals such as RES.

The stepwise regression modeling and ANN performed here were used to assess the relationships between three factors of cultivar, organ and time as well as three total measured factors, i.e., TRC, TFC, and TAA with the contents of phenolics in grapevine foliar parts and the possibility of the prediction of phenolics content according to determined factors. Such mathematical relationships and predictions have not been previously reported by researchers in this area. ANN-based models were compared with stepwise regression models considering accuracy of the prediction, relative importance, and the effect of input variables on phenolics content.

Neural networks are able to learn complex relationships and generalize results from given patterns of input/output data. Therefore, ANNs are appropriate techniques for the modeling of complicated systems for which precise models or probable performances have not been found. Solving a problem using ANNs depends on the magnitude, quality and preprocessing of the training data, type, and construction of the ANN and the learning algorithm for that special case (Baykal and Yildirim, 2013).

The key privilege of ANN-model is that it is not necessary to specify a preceding proper fitting function; so, it has a complete calculation capability to estimate practically all types of non-linear functions which helps us to develop the most accurate prediction model. Based on the high accuracy of the predicted data both in the training and testing processes, we can conclude that the offered neural networks are capable of predicting the respective phenolic content in grape foliage. Despite lots of research reports on the existing correlation between TAA, TRC, or TFC with the individual phenolics in grape berries, leaves, stems, and wine (Bors et al., 1990; Rice-Evans and Miller, 1996; Torres et al., 2015; Eftekhari et al., 2017), there is still this question that which phenolic compounds have direct relationship with the mentioned total indices. As mentioned above, the sensitivity of the studied phenolics prediction models to continuous input variables, i.e., TRC, TFC, and TAA is less than three categorical inputs, i.e., cultivar, time and organ but the ANN-models were constructed considering all inputs together. In our previous work (Eftekhari et al., 2017), we concluded that the type of the phenolic compound is important in determining the antioxidant activity of an extract as it has also been stated by other researchers (Rice-Evans and Miller, 1996). Here, we can more precisely state that OC is more related to the TAA than other investigated phenolics (**Table 4**). The most sensitive flavonoid model to TAA is the model related to QUE which has been previously found as the most powerful flavonoid with the anti-oxidative action (Bors et al., 1990; Rice-Evans et al., 1996). And one of its glycosylated forms, i.e., IQ, as a flavonoid, was also more sensitive to TAA than TFC (**Table 4**).

Previous modeling studies in different research areas have also indicated substantially higher accuracy of ANN modeling technique than regression modeling (Jamshidi et al., 2016) or other modeling procedures (Moghri et al., 2015). Comparing other regression methods performances like partial least squares (PLS) with ANN using spectrum data of near-infrared (NIR) for the prediction of total anthocyanin concentration in red-grape homogenates revealed that the PLS prediction had a high error at concentration extremes while ANN provided a higher correlation (Janik et al., 2007). In order to estimate total phenol in tea, Luo et al. (2005) also used NIR with ANN rather than a linear PLS model theoretically to expand the applicable analysis range as mentioned in the wine study. Furthermore, it has been displayed that GA is an easy, precise and effective optimization method (Moghri et al., 2015) which can be useful for developing an optimized number of neurons in hidden layer of ANN for constructing prediction model of phenolics composition in grapevine foliar parts.

Neural models have been used before in order to assessment of β-carotene and lycopene concentrations in samples of food to solve the intervention of analytical techniques UV-vis and HPLC (Cámara et al., 2010) but these models have not been developed previously for modeling the effect of different factors like cultivar, time and organe on phenolics yield in plants as well as even using TAA, TRC, or TFC indices to predict the presence of phenolics to avoid using complex and expensive analytical techniques like HPLC.

Factors like altitude of the place in which grapevine is growing and the skin color of grape berry can be added as inputs to the model to achieve more extensive models and predictions. Thus, future studies are suggested to evaluate these facts in the grapevines with the aim to establish the effect on the phenolic composition of foliage.

## Conclusion

Twelve ANN-models were constructed to predict targeted phenolics (GAL, CAT, PC, MC, OC, RUT, IQ, COU, RES, QUE, NAR, and KAE) levels in the grapevine foliage. Very small differences between the ANN predicted results and experimental data of the phenolic concentration confirmed the outstanding performance of the GA-ANN method. This can be attributed to the ANN capability to construct non-linear mapping of data. Sensitivity analysis of the ANN-model revealed that the organ was the most important controlling factor affecting the content of each phenolic compound, followed by sampling time, and then cultivar; however, considering the total measured indices as predictive factors, phenolics were most related to TRC, followed by TFC, and then TAA with the exception of OC, QUE, NAR and KAE which were somehow different.

These results are promising from the standpoint of the industrial exploitation of grapevine foliage wastes and can be assumed as a starting point to design future studies focused on the determination of phenolics composition in different parts of the grape including fruit, wine and wastes of industrial processing from different cultivars.

## Author Contributions

All authors listed have made a substantial, direct and intellectual contribution to the work, and approved it for publication.

## Conflict of Interest Statement

The authors declare that the research was conducted in the absence of any commercial or financial relationships that could be construed as a potential conflict of interest.

## Abbreviations

ANN: artificial neural network
CAT: catechin
COU: coumarin
GA: genetic algorithm
GAL: gallic acid
IQ: isoquercitrin
KAE: kaempferol
MC: *m*-coumaric acid
NAR: naringenin
OC: *o*-coumaric acid
PC: *p*-coumaric acid
QUE: quercetin
RES: resveratrol
RUT: rutin
TAA: total antioxidant activity
TFC: total flavonoid content
TRC: total reducing capacity
